# Nutrient control of splice site selection contributes to methionine addiction of cancer

**DOI:** 10.1016/j.molmet.2025.102103

**Published:** 2025-01-23

**Authors:** Da-Wei Lin, Francisco G. Carranza, Stacey Borrego, Linda Lauinger, Lucas Dantas de Paula, Harika R. Pulipelli, Anna Andronicos, Klemens J. Hertel, Peter Kaiser

**Affiliations:** 1 Department of Biological Chemistry, School of Medicine, University of California, Irvine, USA; 2 Department of Microbiology and Molecular Genetics, School of Medicine, University of California, Irvine, USA

**Keywords:** Methionine dependence of cancer, Methionine addiction, Splicing, Prmt5, Methylation

## Abstract

**Objective:**

Many cancer cells depend on exogenous methionine for proliferation, whereas non-tumorigenic cells can divide in media supplemented with the metabolic precursor homocysteine. This phenomenon is known as methionine dependence of cancer or methionine addiction. The underlying mechanisms driving this cancer-specific metabolic addiction are poorly understood. Here we find that methionine dependence is associated with severe dysregulation of pre-mRNA splicing.

**Methods:**

We used triple-negative breast cancer cells and their methionine-independent derivatives R8 to compare RNA expression profiles in methionine and homocysteine growth media. The data set was also analyzed for alternative splicing.

**Results:**

When tumorigenic cells were cultured in homocysteine medium, cancer cells failed to efficiently methylate the spliceosomal snRNP component SmD1, which resulted in reduced binding to the Survival-of-Motor-Neuron protein SMN leading to aberrant splicing. These effects were specific for cancer cells as neither Sm protein methylation nor splicing fidelity was affected when non-tumorigenic cells were cultured in homocysteine medium. Sm protein methylation is catalyzed by Protein Arginine Methyl Transferase 5 (Prmt5). Reducing methionine concentrations in the culture medium sensitized cancer cells to Prmt5 inhibition supporting a mechanistic link between methionine dependence of cancer and splicing.

**Conclusions:**

Our results link nutritional demands to splicing changes and thereby provide a link between the cancer-specific metabolic phenomenon, described as methionine addiction over 40 years ago, with a defined cellular pathway that contributes to cancer cell proliferation.

## Highlights

• Methionine restriction induces dysregulation of splicing in cancer cells.
• Methylation of spleiceosomal snRNP components is hypersensitive to nutrient stress.
• Methionine restriction induces hypersensitivity to Prmt5 inhibitors in cancer cells.

## 1 Introduction

**Figure 1:**
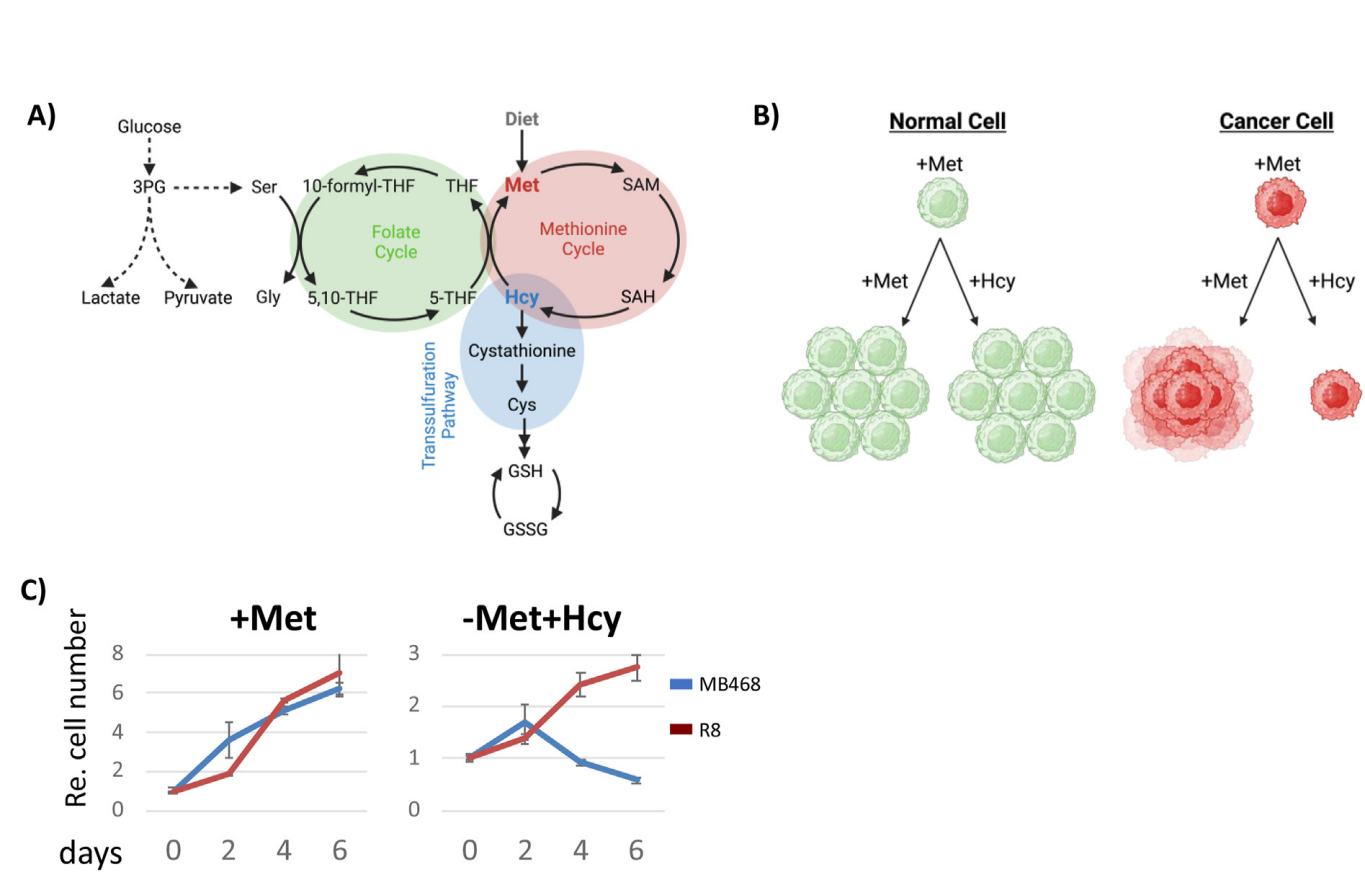
**Methionine dependence in cancer.** (A) Pathways related to one-carbon metabolism. (B) Schematic depiction of methionine addiction of cancer. (C) Methionine-dependent (MDA-MB468) and methionine-independent (R8) cells were cultured in M^+^ or M^−^H^+^ medium for 6 days and cell proliferation was measured.

Cancer cells and tumors display unique metabolic requirements, such as the well-known Warburg effect [[1], [2], [3], [4], [5]]. A less-studied metabolic dependence specific to cancer is the requirement for exogenous methionine [6,7]. Proliferation rates of non-tumorigenic cells are indifferent whether the methionine cycle is fed by exogenous methionine or homocysteine (Figure 1A), but many cancer cells can only efficiently proliferate with exogenous methionine, even though they readily convert homocysteine into methionine (Figure 1B) [6,[8], [9], [10], [11], [12]]. This metabolic dependence was first observed in 1973 using mouse leukemia cells [12], and subsequent experiments extended the methionine/homocysteine substitution studies across a variety of cell lines derived from different tumor types [6,10,11,[13], [14], [15], [16]]. Pluripotent stem cells also show a high requirement for methionine metabolism for maintenance and differentiation [17]. These findings demonstrated that most cancer cells require exogenous methionine, whereas most non-tumorigenic cells are not affected by the replacement of methionine with homocysteine (Figure 1B). This phenomenon is commonly referred to as the methionine dependence of cancer, methionine-stress sensitivity of cancer, methionine addiction, or “Hoffman effect”. These terms might be confusing, as all cells require methionine for growth, and methionine stress could be mistaken for a condition caused by high methionine levels. However, these terms are used to specifically indicate that replacing exogenous methionine with homocysteine in growth media inhibits the growth of many cancer cells, whereas non-tumorigenic cells generally remain unaffected. Methionine is an essential amino acid, prompting all cells to activate adaptive pathways under methionine-depleted or methionine-reduced conditions. However, supplementation with homocysteine can overcome these effects in non-tumorigenic cells and imposes dependence on exogenous methionine specifically on cancer cells.

This cancer-specific metabolic phenomenon is also reflected in hematological cancers and solid tumors because reducing available methionine can slow tumor progression in animal models and significantly enhance response to chemotherapy [[18], [19], [20], [21], [22]]. It soon became clear that the addiction of cancer cells to exogenously provided methionine is not due to their failure to synthesize methionine from homocysteine [10], and experiments suggested an increase in transmethylation reactions in cancer cells that outstrips regeneration as the underlying metabolic cause [23,24]. Furthermore, metabolic tracing experiments identified a reduced capacity of cancer cells to synthesize the primary methyl-donor S-adenosylmethionine (SAM) when grown in homocysteine medium [8,15,25]. To compensate for reduced SAM production, cancer cells seem to divert co-factors and precursors necessary for nucleotide and glutathione biosynthesis, which results in reduced nucleotide and antioxidant availability [8,21,26]. In addition, lipid metabolism is significantly affected in cancer cells when cultured in homocysteine, although this alteration may not have a direct effect on cancer cell proliferation [27]. Despite this progress molecular pathways that connect these altered metabolic circuits to cell proliferation and explain methionine dependence of cancer remain to be identified.

The elevated requirement for SAM in cancer cells appears to be central to the link between methionine dependence and cell cycle progression, as supplementing homocysteine-containing growth media with exogenous SAM mitigates the dependency on methionine [9,15,23]. It is not surprising that the one-carbon cycle, which resynthesizes SAM from methionine and ATP to sustain the cellular methylation potential, is connected to cancer cell proliferation. The one-carbon cycle connects to several pathways that are crucial for cell proliferation, such as nucleotide metabolism via the folate cycle, antioxidant production through glutathione synthesis, and polyamine synthesis (Figure 1A) [28]. The importance of this metabolic pathway for cell proliferation is further emphasized by an evolutionary conserved dedicated cell cycle checkpoint that monitors methionine and SAM metabolism from yeast to humans [15,[29], [30], [31]]. The reason for the increased dependence of cancer cells on methionine and SAM is not well understood, but connections to glutathione synthesis, the folate cycle, polyamine production, and increased demand for transmethylation are likely contributors to methionine addiction [6,26]. Molecular mechanisms that contribute to proliferation arrest and cell death when cancer cells are cultured in homocysteine media are just beginning to emerge.

We demonstrate that splicing fidelity is significantly disrupted in cancer cells grown in homocysteine medium. Using the triple-negative breast cancer cell line MDA-MB-468 and its methionine-independent derivative, R8, we leverage a well-characterized metabolic and cell cycle model. Additional cell systems corroborate our findings, linking metabolic influences on splicing fidelity to methionine addiction in cancer. Cancer cells frequently display changes in splicing outcomes, many of which have the potential to contribute to disease [32]. For example, analyses of thousands of tumor samples across 32 cancer types uncovered widespread tumor-specific alterations in mRNA isoform formation [33,34], demonstrating that splicing alterations represent an important molecular feature of cancer biology [35]. These splicing changes are often linked to mutations in splicing-regulatory elements or core components and regulators of the splicing machinery [35,36]. In addition, it is appreciated that the proliferative nature of cancer cells imposes high demands on the splicing machinery, thereby sensitizing cancer cells to splicing perturbations [37].

In this study, we mechanistically link cancer-specific nutritional demands to splicing fidelity.

## 2 Material and methods

### 2.1 Cell culture, cell proliferation, and cell viability

Cell lines were maintained in DMEM (Sigma–Aldrich, D0422) supplemented with 10 % dialyzed fetal bovine serum (FBS, Omega Scientific), 1.5 μM cyanocobalamin (vitamin B12), 4 mM l-glutamine, 100 μM l-cysteine (Fisher Scientific), and 100 μM l-methionine (Sigma–Aldrich). The growth media contained 4 mg/L each folic acid and choline chloride. In the case of methionine-free media, 370 μM dl-homocysteine (Sigma–Aldrich) was added in the absence of methionine. Cell proliferation was determined in 96-well plates using the CellTiter-Glo reagent (Promega) according to manufacturer's instructions. Luminescence was measured on a Clariostar plate reader in quadruplicate samples (BMG Labtech). The fraction of viable cells was determined in 96-well plates using CellTox Green reagent (Promega) according to manufacturer's instructions. Luminescence was measured on a Clariostar plate reader in sextuplicate samples (BMG Labtech).

### 2.2 Detection of reactive oxygen species

MDA-MB460 and R8 ells were grown in methionine-containing media and switched to homocysteine media for 4, 8,12, and 24 h. MDA-MB468 cells in methionine medium were treated with 500 μM H_2_O_2_ for 30min as a positive control.

Cells were harvested and treated with 5-(and-6)-chloromethyl-2′,7′-dichlorodihydrofluorescein diacetate, acetyl ester (CM-H2DCFDA) (Thermo Fisher C6827) at a final concentration of 10 μM for 10 min in the same M^+^ or M^−^H^+^ culture medium, except 2% dialyzed FBS was used. Subsequently, 10,000 cells, gated for single cells, were analyzed by flow cytometry to detect fluorescence of the oxidized CM-H2DCFDA in the FITC channel. 3 biological replicates were analyzed.

### 2.3 Splicing reporter assay

Cells were grown in 6-well plates transfected with 2 μg of either Luc (CMV-Luc2CP/ARE, Addgene #62857) or Luc-I (CMV-LUC2CP/intron/ARE, Addgene #62858). Cells were shifted to control (+M) or homocysteine (-Met + Hcy) media conditions 24 h post-transfection. After 2 days of incubation cells were harvested, counted, and 10,000 cells were resuspended in 100 μl of the appropriate media. The cell suspension was then combined with 100 μl of ONE-Glo™ Luciferase Assay System reagent. After a 10-minute incubation period, the luminescent signal was measured using a luminometer.

### 2.4 SMN immunopurification

MB468 and R8 cells were grown in a 10 cm dish in media containing methionine or homocysteine respectively. Cells were mildly crosslinked for 10 min at RT under shaking with 0.05% Formaldehyde. Media was removed and cells were trypsinized and gently scaped off plate with a rubber scraper, pelleted and washed with ice cold 1xPBS. The pellet was resuspended in NET Buffer (150 mM NaCI, 50 mM Tris-HCI [pH 7.4], 5 mM EDTA [pH 8], 1% NP-40, 10% glycerol, 1 mM phenylmethylsulfonyl fluoride [PMSF], 1 mg/ml each leupeptin and pepstatin, 10 mM Na-pyrophosphate, 50 mM NaF and 0.1 mM orthovanadate). Samples were incubated for 60 min at 4 °C on a nutator. Lysates were cleared at 12k rpm at 4 °C for 30 min and concentrations were determined by OD_280_. 500 μg of lysate was used per IP and filled up to 500 μl with NET buffer. 2 μg of SMN antibody (Santa Cruz, sc-32313) was added and incubated overnight at 4 °C on a nutator. The next day samples were added to 20 μl of NET buffer equilibrated Protein G Sepharose slurry and incubated for 2.5h on a nutator at 4 °C. Beads were washed 3x in 1 ml cold NET buffer and finally resuspended in 2x SDS-PAGE buffer and incubated for 5 min at 95 °C. Samples were analyzed by SDS-PAGE followed by Western blot using the SMN and SmD1 (Abcam, ab50940) antibody respectively. The results shown are representative blots from three independent experiments.

### 2.5 Gene expression analysis

Total RNA was isolated from MB468 and MB468res-R8 cells grown in the indicated growth conditions. RNeasy Plus Mini Kit (Qiagen, Cat No. 74134) was used for extraction and library preparation was performed using the TruSeq RNA Library Preparation Kit v2 (Illumina, RS-122-2001 and RS-122-2002) with the ERCC RNA Spike-In mix (ThermoFisher, 4456740) to control for sample preparation variation. PolyA-selected libraries were generated and sequenced at the Genomics Research and Technology Hub (GRT Hub) at the University of California, Irvine Genomics High Throughput Facility on a HiSeq 4000 system using single-end 100bp reads.

### 2.6 Bioinformatic analysis

Raw reads were aligned to a custom human genome, GRCh38/hg38, using the UCSC Genome Browser and the ERCC spike-in sequences (http://tools.invitrogen.com/downloads/ERCC92.fa) using HISAT2 and STAR alignment software [38,39]. The number of reads mapped to each gene feature was quantified by featureCounts in the Rsubread package, and unwanted sample variation was determined by RUVSeq [40,41]. Differential gene expression analysis was performed using DESeq2 with featureCounts files [42]. The DESeq2 parameters used were FDR of 0.01 and fold-change difference of 2. Pathway enrichment analyses were conducted using the Shiny GO Enrichment analysis tool [43]. PCA plots and Venn diagrams were generated using a custom R script. Alternative splicing analysis was carried out using rMATS [44]. rMATS is a statistical program designed to detect differential alternative splicing between two RNA-Seq samples. The rMATS parameters used were an FDR cut-off of 0.05 and ΔPSI (differential percent spliced-in) of 10%. Differential expression volcano plots were created using the R tool “EnhancedVolcano” (https://github.com/kevinblighe/EnhancedVolcano). Global splicing effect volcano plots were generated using the MASER R package (https://github.com/DiogoVeiga/maser).

## 3 Results

### 3.1 Expression profile changes in MDA-MB468 cells in response to methionine depletion

We have previously developed an isogenic methionine-dependent and independent cell pair based on the triple-negative breast cancer cell line MDA-MB468 (Figure 1C) [8,15,45]. Such isogenic cell models have originally been developed by Hoffman and colleagues using SV40 transformation of fibroblasts [46,47] and later expanded to other cell pairs by a selection process to revert the addiction of cancer cells on exogenous methionine [15,45,48,49]. Similar to other such selected cell pairs, we do not know the changes responsible for the reversion of MDA-MB468 cells to methionine-independent R8 cells. However, we selected the MDA-MB468/R8 cells for these studies because they are the best-characterized model with extensive cell cycle and metabolic characterization [8,15,27]. It is important to note that excess homocysteine as we use in the following experiments has been linked to oxidative stress [50]. However, during the timeframe of our studies, we did not detect any increase in reactive oxygen species when cells were shifted to homocysteine growth media excluding indirect effects through induced oxidative pressure (Figure S1A).

**Figure 2:**
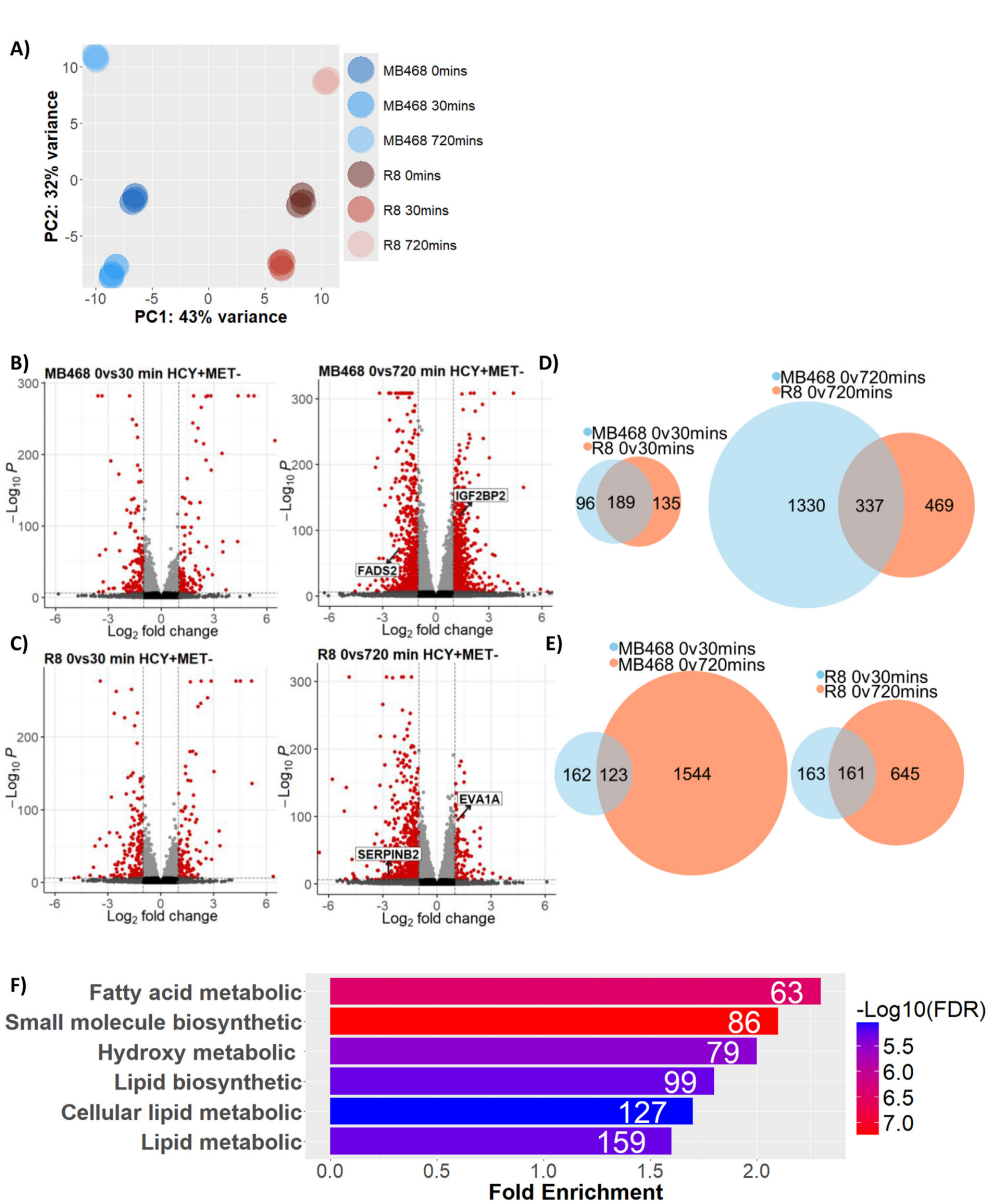
**RNA expression response to methionine/homocysteine switch.** (A) Principal component analysis indicating MDA-MB468 and R8 cell lines have distinct expression profiles but respond similarly to methionine restriction. Differential expression of genes 0, 30, and 720 min into methionine/homocysteine switch for MDA-MB468 (B), or R8 (C) cells. (D) Ven diagrams illustrating common differentially expressed genes across cell lines at the same time after methionine restriction. (E) Ven diagrams illustrating common differentially expressed genes within cell lines at different time points into methionine restriction. (F) Ontology terms of differentially expressed genes in MDA-MB468 cells 720 min after methionine withdrawal.

We cultured the MDA-MB468/R8 cell pair in methionine-containing medium (M^+^) and then shifted to methionine-free medium supplemented with homocysteine (M^−^H^+^) for 30 min to capture the immediate transcriptional response, and 12 h to evaluate long-term changes. The significance cut-off for differential gene expression was set to p < 0.01 for all analyses. Principal component analyses separated the two cell lines in various clusters indicating not only a distinct expression profile in M^+^ medium but also a relatively minor response after culturing for 30 min in M^−^H^+^ medium that was amplified after 12 h (Figure 2A–C). R8 cells are derived from MDA-MB468 by selecting for growth in M^−^H^+^ medium. Gene ontology analyses of differentially expressed genes in methionine medium between the two cell lines did not reveal any distinct biological processes that could explain their different metabolic needs (Figure S1B). Expression changes after 30 min in M^−^H^+^ medium were comparable between MDA-MB468 and R8 cells (Figure 2B–D, left panels), with mainly RNA Polymerase II-related processes enriched S1C). By contrast, the two cell lines responded very differently after 12 h in M^−^H^+^ growth conditions (Figure 2B–D, right panel, and 2E). Gene enrichment identified lipid metabolism as the unique up or down-regulated processes for methionine-addicted MDA-MB468 cells (Figure 2F). This is consistent with a previous system-wide lipid profiling study that linked changes in lipid metabolism to ER stress and methionine dependence [27]. We also compared the trend of gene expression changes from 30 min to 12h in M^−^H^+^ medium. In both cell lines expression changes increased significantly, with most genes induced in the later time point and methionine-dependent cells showing a much more pronounced modulation of gene expression programs as compared to the methionine-independent R8 cells (Figure 2E). While significant remodeling of gene expression occurs when cells are shifted from methionine-to homocysteine-containing growth media, affected genes did not suggest a connection to cell proliferation or other processes that could provide mechanistic insight into communication between methionine metabolism and cell cycle regulation.

### 3.2 Exogenous methionine is important for splicing fidelity in methionine-addicted cancer cells

Results from expression profiling generated some insight into biological processes affected by methionine addiction, but very few changes suggested a link to cell proliferation and thus a molecular mechanism for how methionine dependence impacts cell cycle regulation. We next analyzed the data for potential effects of methionine dependence on splicing. Synthetic lethality has previously been identified between the deletion of S-methyl-5′-thioadenosine phosphorylase (MTAP), a key enzyme in the methionine salvage pathway, and knockdown of the arginine methyltransferase Prmt5. This genetic interaction was connected with aberrant splicing through a mechanism that proposed the accumulated substrate of MTAP, methylthioadenosine, as a competitive inhibitor of Prmt5 [51], and Prmt5 has been known to regulate splicing fidelity [[52], [53], [54]]. Given the metabolic connection of MTAP with the one-carbon cycle we explored a possible link between splicing and methionine addiction.

**Figure 3:**
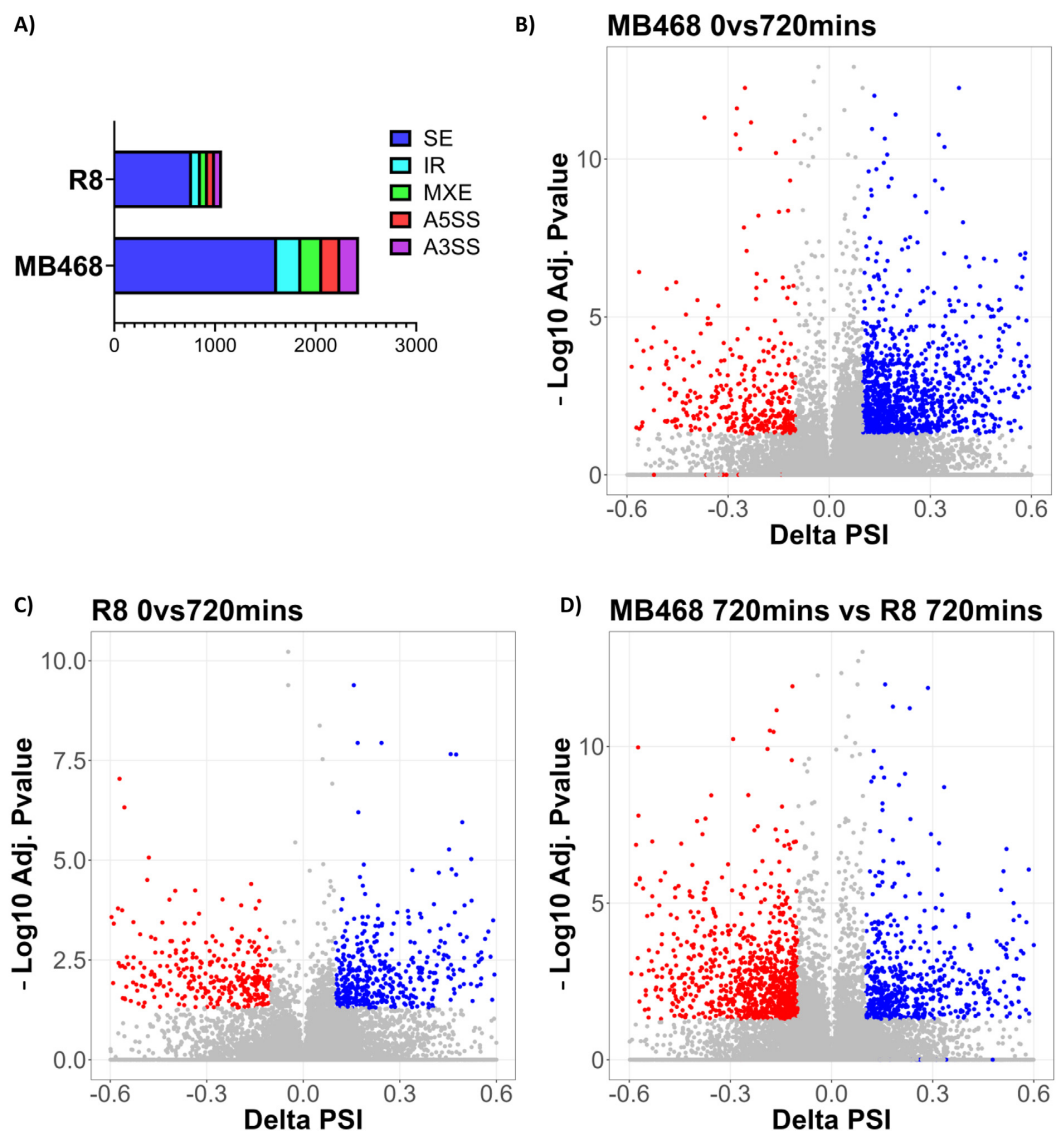
**Methionine restriction in MDA-MB468 cells impacts splicing fidelity.** (A) A stacked bar graph displaying MDA-MB468 and R8 alternative splicing event distributions 720 min into M^−^H^+^ medium shift. SE: skipped exons; IR: intron retention; MXE: mutually exclusive exons; A5SS: alternative 5′ splice sites; A3SS: alternative 3′ splice sites. (B and C) Volcano plots displaying the difference in percent spliced in (PSI) of skipped exon events in MDA-MB468 (B) or R8 (C) cells upon switch to homocysteine growth media. Blue indicates higher exon inclusion levels before methionine restriction. Red indicates higher inclusion levels after methionine restriction. (D) Volcano plot displaying differential exon inclusion events in MDA-MB468 and R8 cells after 729 min of methionine restriction.

To determine if alternative splicing changes are observed upon shifting cells to M^−^H^+^ medium the gene expression data from the 0- and 12-hour timepoints were analyzed using rMATS, a computational tool designed to detect local changes in exon inclusion levels. The analysis identified 2,429 alternative splicing events in MDA-MB468 cells (MDA-MB468-M^-^H^+^) and 1,069 alternative splicing events in R8 cells (R8-M^-^H^+^) (Figure 3A). Interestingly, alternative exon inclusion is the most common MDA-MB468 alternative splicing event (1,616, FDR <0.05, inclusion level difference ± 0.10), of which ∼75% are associated with reduced exon inclusion after the switch to the M^−^H^+^ medium (Figure 3B). These observations suggest that the recognition of alternative exons in MDA-MB468 is generally reduced upon methionine/homocysteine switch. An analogous time course analysis of the methionine-independent R8 cells identified 773 statistically significant exon inclusion events, more evenly distributed between increased (42%) and reduced (58%) exon skipping (Figure 3C). To determine splicing changes related to methionine dependence we compared splice patterns between MDA-MB468 and the methionine independent R8 cells after 12 h of methionine withdrawal (MDA-MB468-M^-^H^+^ vs R8-M^-^H^+^). Consistent with the notion that the presence of methionine promotes exon inclusion, 60% of the splicing events detected are characterized by lower exon inclusion levels in MDA-MB468 cells when compared to R8 cells (Figure 3D). Several of the identified alternative splicing events were verified using exon-specific PCR (Figure S2) and their alternative splicing patterns based on deep sequencing are illustrated using Sashimi plots (Figure S3).

**Figure 4:**
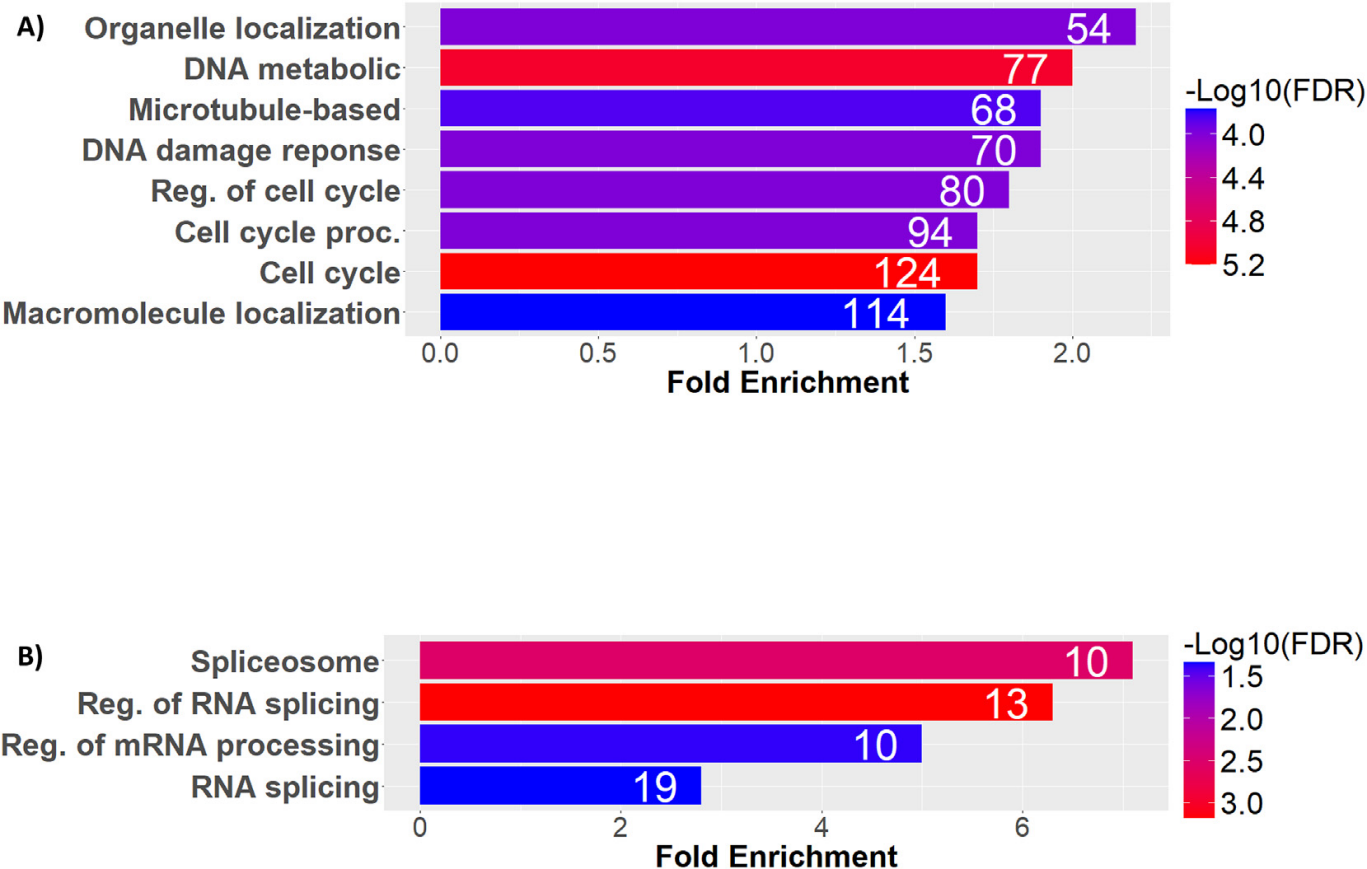
**Gene ontology analysis of alternatively spliced genes upon methionine/homocysteine switch in MDA-MB468 cells.** (A) Significantly enriched biological processes of genes associated with increased exon skipping upon methionine withdrawal in MDA-MB468 cells. (B) Significantly enriched biological processes of genes associated with decreased exon skipping upon methionine withdrawal in MDA-MB468 cells. The numbers in the bars represent the number of genes hits in each category. The colors indicate statistical significance.

Pathway analysis was used to determine what biological processes might be affected by MDA-MB468's dependence on exogenous methionine. Genes characterized by exon skipping upon methionine withdrawal in MDA-MB468 cells fall into general categories of cell cycle, mitotic cell cycle, regulation of cell cycle, DNA repair, and positive regulation of DNA metabolic processes (Figure 4A). These terms indicate an association between pre-mRNA splicing changes upon the methionine/homocysteine switch and cell division. Interestingly, genes characterized by increased exon inclusion during methionine withdrawal are categorized into RNA splicing and regulation of RNA splicing processes (Figure 4B). These observations suggest that alternative splicing of RNA processing-related factors mediate reduced inclusion of exons within cell cycle control genes, thereby inhibiting proliferation.

We extended the splicing analyses to another methionine-dependent cell line, HEK293T, chosen for its oncogenic transformation by the SV40 large T antigen (Figure 6C). RNA-seq data from cells cultured in M^+^ and M^−^H^+^ media for 12 h were compared. Similar to MDA-MB468 triple-negative breast cancer cells, the shift to homocysteine-containing media triggered widespread alternative splicing events (Figure S4A). Notably, there was a significant overlap in the altered splicing events between HEK293T and MDA-MB468 cells (Figure S4B). Importantly, as observed in MDA-MB468 cells, the affected genes in HEK293T cells predominantly belonged to categories involved in cell cycle regulation, DNA replication, DNA repair, and general stress response (Figure S4C). These findings further validate that methionine-dependent cells undergo extensive alternative splicing upon exposure to homocysteine media, primarily impacting genes critical for cell cycle progression.

**Figure 5:**
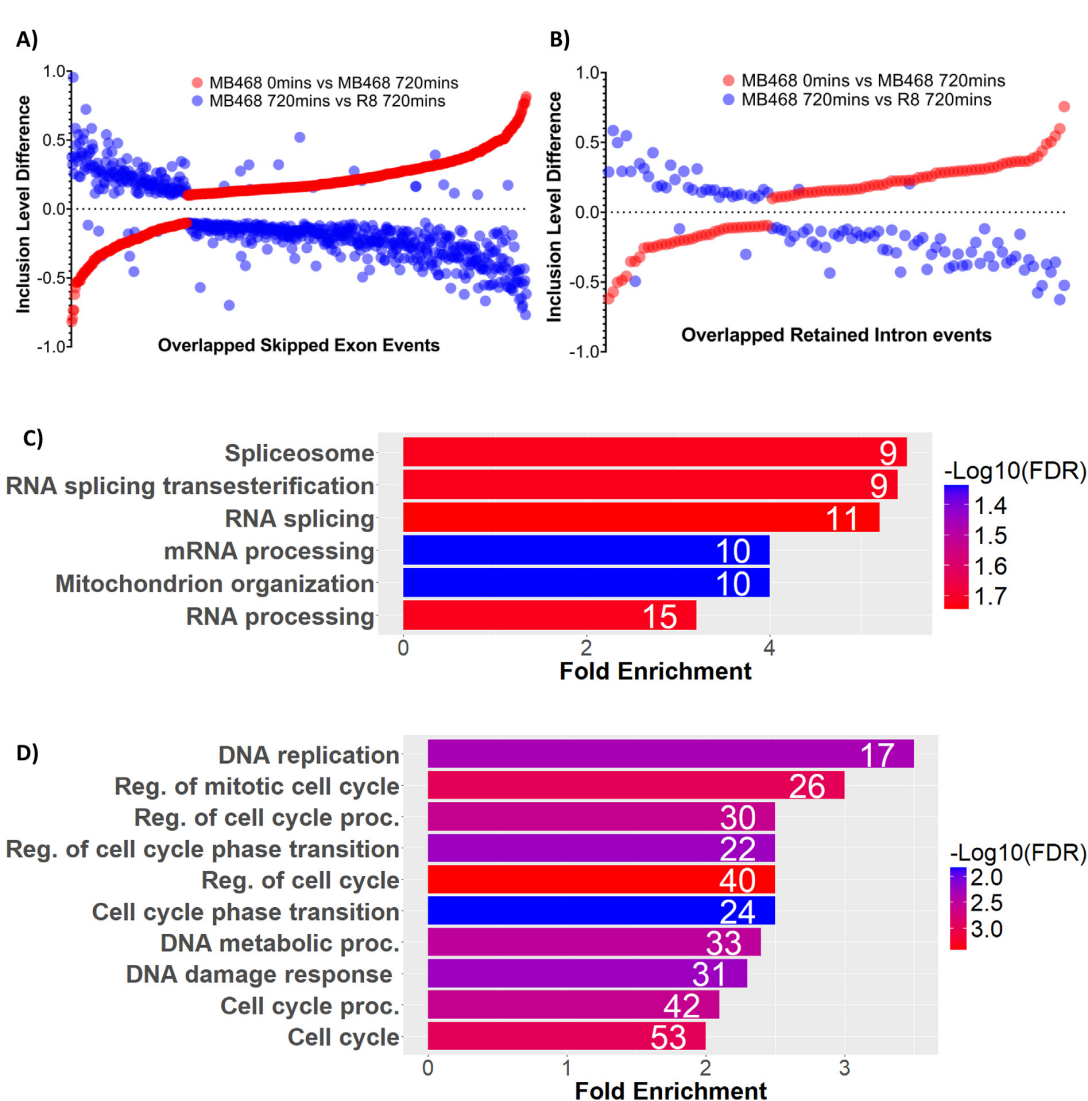
**Inverse relationship between overlapping skipped exon and intron retention events in MDA-MB468 and R8 cells.** (A) Each dot in the graph represents an overlapping exon inclusion or exclusion event, organized by the inclusion difference observed upon methionine withdrawal in MDA-MB468 cells (red dots). The blue dots represent the difference observed in identical exon inclusion events when MDA-MB468 cells are compared with R8 cells after 12 h in M^−^H^+^ medium. The y-axis displays the event inclusion level difference. The x-axis displays an overlapping differential alternative splicing event. (B) Plot displaying overlapping alternatively retained intron events for the same datasets as described in (A). (C) Gene ontology analysis of overlapping alternative splicing events (A) that display increased exon skipping in MDA-MB468 cells after methionine withdrawal. (D) Gene ontology analysis of alternative splicing events (A) that display increased exon retention in MDA-MB468 cells after methionine withdrawal. The numbers displayed in the bar graphs represent the number of gene hits in each category. The colors indicate statistical significance.

**Figure 6:**
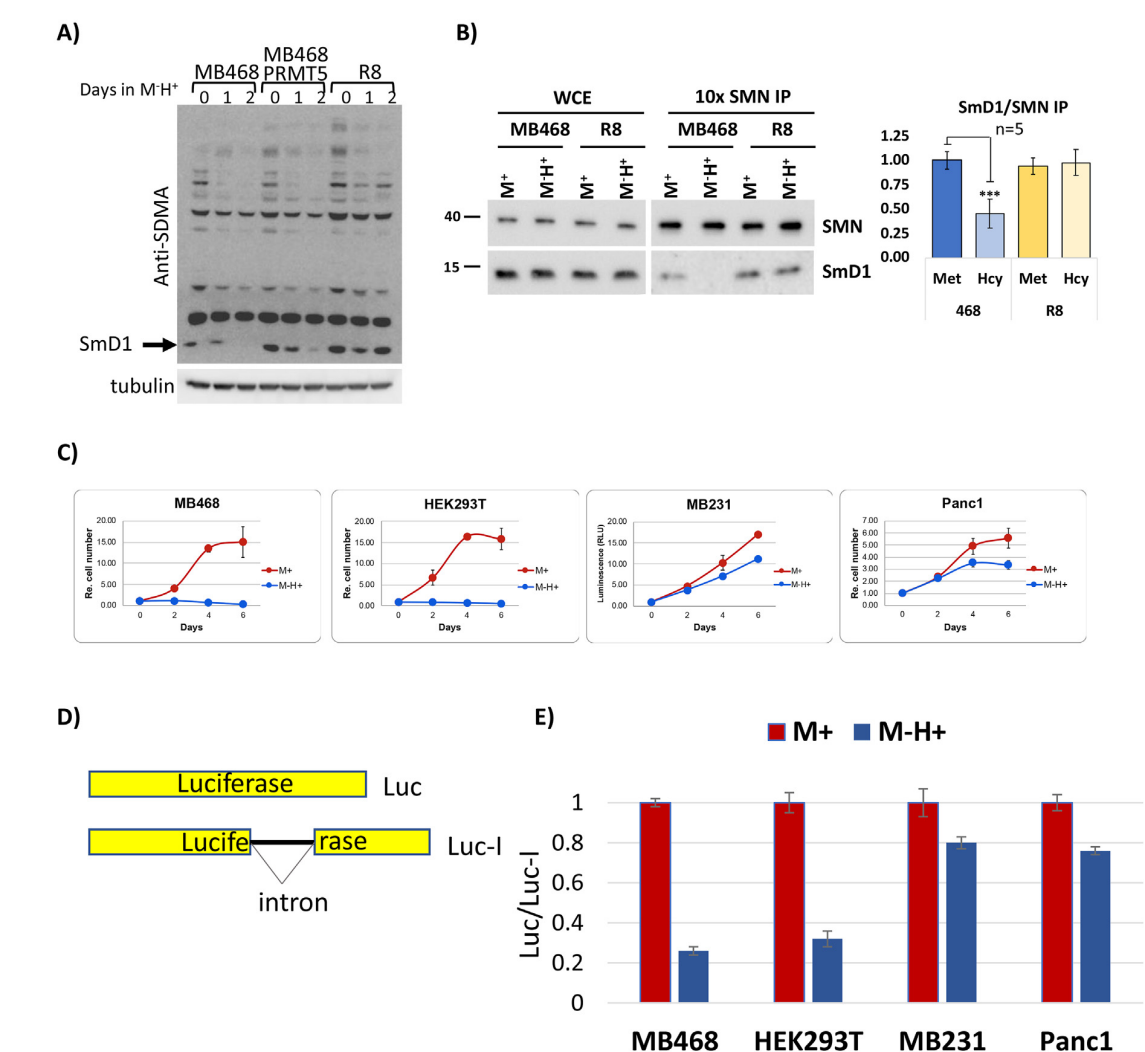
**Methionine/homocysteine media switch of MDA-MB468 cells leads to a loss in SmD1 methylation and splicing fidelity.** (A) Western blot using anti-SDMA (symmetrical dimethylarginines) antibodies (left) in MDA-MB468, MDA-MB468 with PRMT5 overexpression, and R8 cell lysates after M^−^H^+^ medium shift. Tubulin serves as a loading control. (B) SMN immunoprecipitation of SmD1 in MDA-MB468 and R8 cells in M^+^ or M^−^H^+^ media. The bar graph to the right represents the quantification of SmD1 band intensities (∗∗∗P ≤ 0.001). (C) Cell growth of methionine-dependent (MB468, HEK293T) and independent (MB231, PANC1) cells in M^+^ and M^−^H^+^ media. (D) Luciferase splicing reporter scheme. Luc refers to the intronless luciferase reporter and Luc-I refers to the intron-containing reporter. (E) Quantitation of relative luciferase activity in different medium conditions in cell lines defined by the x-axis (n = 4, +/− STD).

If methionine addiction is a major driving force in splicing differences, it is likely that specific exons skipped in MDA-MB468 cells are retained at higher rates in R8 cells upon methionine restriction. This is indeed the case. Figure 5A illustrates the inclusion level difference of two alternative splicing comparisons merged on top of each other based on shared differentially included exon events. Alternative exon skipping induced in MDA-MB468 cells upon methionine withdrawal (MDA-MB468-M^-^H^+^) and exon skipping differences across both cell lines upon methionine withdrawal (MDA-MB468-M^-^H^+^ vs R8-M^-^H^+^) displayed 645 shared differential exon splicing events. Interestingly, events that resulted in reduced exon inclusion in MDA-MB468 cells upon methionine withdrawal were retained in R8 cells (Figure 5A). The inverse correlation is also observed for exons that displayed increased exon inclusion levels in MDA-MB468 cells upon methionine removal. A gene ontology analysis of these overlap events focusing on increased skipped exons in MDA-MB468 cells linked the affected genes to RNA processing (Figure 5C) while the overlap events focusing on increased exon retention were linked to cell cycle processes (Figure 5D). These results suggest that dependence on exogenous methionine in MDA-MB468 cells results in different mRNA isoform expression of genes that regulate pre-mRNA splicing and genes associated with cell division.

A complementary intron retention analysis also highlights genes associated with RNA processing, RNA splicing, and the regulation of RNA processing. These observations further support the notion that methionine addiction leads to alternative splicing in genes involved in pre-mRNA splicing, potentially contributing to mis-splicing antagonistic to cell proliferation. Remarkably, as was observed for exon skipping, splicing events with increased intron retention upon methionine withdrawal in MDA-MB468 cells overlapped with decreased intron retention events in R8 cells (Figure 5B). Thus, the same splicing events that resulted in increased intron retention after methionine withdrawal in MDA-MB468 cells are more efficiently spliced out in methionine-independent R8 cells. The results from the overlap comparisons provide evidence that methionine dependency in cancer cells leads to pre-mRNA dysregulation, which may be detrimental to cell proliferation.

### 3.3 Methionine-dependent cancer cells fail to efficiently methylate Sm proteins

The in-depth analyses of genome-wide splicing in methionine-dependent MDA-MB468 cells and the methionine-independent R8 derivatives indicated that fidelity of splicing is affected by changes in exogenous methionine supply. Importantly, splicing in methionine-independent cells was much less affected by these metabolic changes. It was previously shown that a key aspect of methionine addiction is the stability of cellular methylation potential during metabolic changes [8,23]. Consistent with that observation, the growth of MDA-MB468 cells in homocysteine medium could be restored in a dose-dependent manner by supplementation with SAM [15]. Prmt5 catalyzes symmetric dimethylation of arginine residues in proteins and this activity has been linked to the regulation of splicing [[52], [53], [54]]. We therefore used a PAN dimethyl-arginine antibody to probe cell lysates for changes in this modification upon replacing methionine with homocysteine in the growth media. Relatively few changes in dimethyl-arginine methylation patterns were observed upon shifting cells to growth medium where methionine was replaced by homocysteine (M^−^H^+^) (Figures 6A, and S5A). However, one prominent band corresponding to the methylated form of the core spliceosome component SmD1 specifically disappeared in methionine-dependent MDA-MB468 cells, while only a transient reduction in the methionine-independent R8 derivatives was observed. This change in methylation pattern caught our attention because SmD1 is a substrate of Prmt5 [55]. Accordingly, overexpression of Prmt5 delayed demethylation of SmD1 in MDA-MB468 cells. SmD1 methylation is thought to enhance binding to the Survival of Motor Neuron (SMN) protein, which contributes to the assembly of the splicing machinery. We, therefore, tested whether methionine dependence leads to reduced interaction between SMN and SmD1 in MDA-MB468 cells. Immunopurification of SMN from cells grown in either methionine or homocysteine-containing media demonstrated that SmD1 binding to SMN was significantly reduced when MDA-MB468 cells were shifted to homocysteine medium (Figure 6B). Methionine-independent R8 cells maintained the SMN/SmD1 interaction under both culture conditions, consistent with unaffected SmD1 methylation in R8 cells (Figure 6A).

### 3.4 Methionine addiction results in reduced splicing activity

We next compared the effect of exogenous methionine on general splicing activity using a luciferase-based splicing reporter in methionine-dependent and independent cells [56]. This reporter was used as a sensitive assay that allows direct measurements of differential splicing efficiencies as intron removal is required for luciferase expression (Figure 6D). This is an engineered reporter frequently used in the field to address whether the efficiency of splicing is altered at evaluated conditions. The intron inserted into the luciferase reporter is 133 nucleotides long and its splice sites are of good strength (5'ss MES = 10.8, 3′ ss MES = 12.4). Thus, this intron should be removed efficiently unless the spliceosome is significantly restricted.

Methionine-dependent (MDA-MB468, HEK293T) and independent cells (MDA-MB231, PANC1) (Figure 6C) were shifted to either M^+^ or M^−^H^+^ medium for 2 days and splicing was monitored by luciferase production normalized to the same cells expressing luciferase without intron (Figure 6D). Methionine-dependent cells had significantly less mature luciferase when cultured in M^−^H^+^ medium as compared to M^+^ medium. By contrast, methionine availability did only modestly affect splicing activity in cells independent from exogenous methionine (Figure 6E). Note that the observed reduced splicing activity is not a reflection of cell viability. Although the cell number increase was minimal in MDA-MB468 and HEK293T once cells were shifted to M^−^H^+^ media, the fraction of viable cells was unchanged over 4 days as compared to cells in methionine media (Figure S5B). The reduced cell proliferation during this period in homocysteine medium is mainly caused by a rapidly induced cell cycle arrest as previously shown [15,31]. These results further confirm that methionine-dependent cells require exogenous methionine to maintain spliceosome activity.

### 3.5 Reduced methylation activity of Prmt5 contributes to methionine addiction

**Figure 7:**
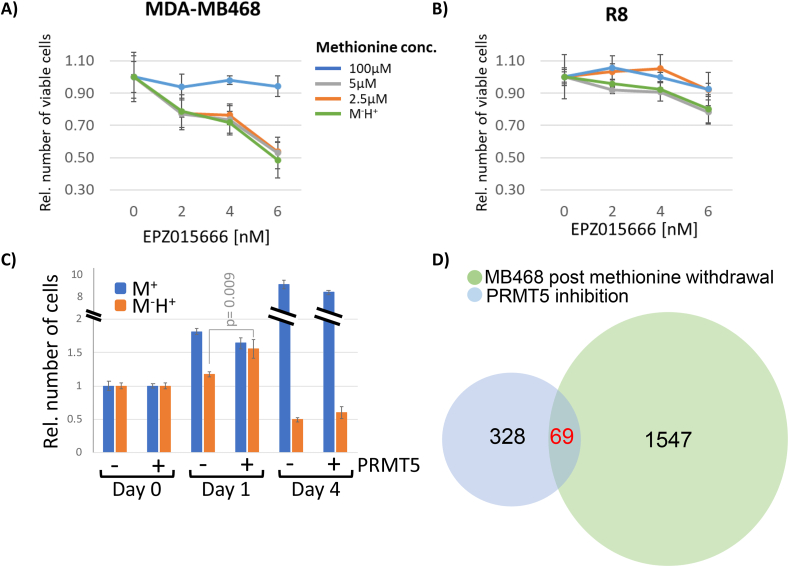
**Decreased methionine availability coupled with PRMT5 inhibition promotes cell death.** (A) The relative number of viable MDA-MB468 cells upon treatment with increased PRMT5 sublethal inhibitor (EPZ015666) concentrations, coupled with variable concentrations of exogenous methionine. (B) Same as in (A) except the cell line R8 was used. (C) Cell proliferation assay in MDA-MB468 cells and MDA-MB468 cells overexpressing PRMT5 in M^+^ or M^−^H^+^ media. (D) Alternative splicing event overlap between MDA-MB468 methionine restriction analysis and an alternative splicing analysis of patient-derived glioblastoma cancer stem cell lines treated with a PRMT5 inhibitor [52].

Our results demonstrate a link between the cancer-specific nutritional requirement for exogenous methionine and modulation of spliceosome activity through inefficient Prmt5-mediated methylation of splicing factors such as SmD1. Whether this nutritional effect contributes to the inability of methionine-dependent cancer cells to proliferate in homocysteine medium, is unclear. This is an important question because other metabolic effects that are closely associated with methionine dependence did not contribute to the cell proliferation defects in methionine-dependent cells [8,27]. We used the specific Prmt5 inhibitor EPZ015666, which has previously been shown to efficiently inhibit Prmt5 activity in MDA-MB468 cells with 20,000-fold selectivity over other protein methyltransferases [[57], [58], [59]]. We reasoned that a synergistic effect of Prmt5 inhibition and availability of exogenous methionine, specifically in MDA-MB468 but not the methionine-independent R8 cells, would strongly support a contribution of Prmt5 activity to cell proliferation defects associated with methionine addiction. Indeed, both restricting exogenous methionine to 5 or 2.5 μM, as well as growth in homocysteine medium sensitized MDA-MB468 cells to sublethal concentrations of the Prmt5 inhibitor (Figure 7A). By contrast, EPZ015666 sensitivity of R8 cells was hardly affected by the different culture media conditions (Figure 7B). These results support that cell proliferation defects associated with methionine addiction are linked to nutrient-impaired methionine metabolism and Prmt5 function. We next asked whether these nutrient-induced effects on Prmt5 present a major pathway that links cell proliferation defects in homocysteine medium to methionine-dependence of cancer. We overexpressed Prmt5 in MDA-MB468 cells and tested their ability to proliferate in homocysteine medium. Prmt5 overexpression initially significantly suppressed the methionine dependence as cells continued to proliferate at day 1 after the shift to homocysteine medium at a rate similar to cells cultured in methionine media (Figure 7C). However, proliferation could not be sustained, indicating that increased Prmt5 expression is not sufficient to overcome methionine addiction. The initial suppression of proliferation defects in homocysteine medium by Prmt5 overexpression is consistent with the synergy between methionine dependence and Prmt5 inhibition (Figure 7A,B) and supports an important role of Prmt5-controlled splicing fidelity in this metabolic addiction. As expected, these initial effects could not be maintained because additional physiological consequences associated with methionine dependence, with possibly later onset, such as changes in metabolic networks, ER stress signaling, reduced defense against oxidative stress, and epigenetic changes can impair cancer cell viability in homocysteine-containing culture media [8,21,27,60,61].

If decreased exogenous methionine concentration or growth in homocysteine medium leads to higher sensitivity towards PRMT5 inhibition and cell proliferation defects, the alternative splicing events triggered by methionine restriction may be the same alternative splicing events observed upon PRMT5 inhibition. Taking advantage of a previously published RNA-seq dataset evaluating the splicing outcome of PRMT5 inhibition in a patient-derived glioblastoma cell culture system [52], we carried out splicing event overlap analyses with our RNA-seq datasets (‘PRMT5 inhibition’ and ‘MDA-MB468-M^-^H^+^’ analysis). Despite the differences in cell lines, growth conditions, and library generation, a statistically significant overlap of 69 events between the two datasets was observed (P < 3.8e^−33^) (Figure 7D). In other words, 21% of all splicing events affected by PRMT5 inhibition in patient-derived glioblastoma cells are also misregulated in breast cancer cells shifted to homocysteine medium. This highly significant alternative splicing event overlap between the datasets suggests that PRMT5 inhibition and methionine dependence impact similar gene pathways and splicing factors that promote cell proliferation defects.

## 4 Discussion

Methionine dependence of cancer has been known for over 40 years, yet cellular pathways that contribute to this common metabolic addiction of cancer cells are only beginning to be identified. Perturbation of metabolic pathways such as cytosolic folate synthesis and mutations or epigenetic changes in genes involved in methionine metabolism have been linked to dependence of exogenous methionine. Most of these alterations contribute but are not sufficient to induce methionine dependence [62,63]. Metabolic profiling and metabolite add-back experiments suggested that methionine-dependent cancer cells experience reduced methylation potential due to decreased SAM/SAH ratios when cultured in homocysteine medium [8,15]. SAM supplementation can mitigate dependence on exogenous methionine [15]. These findings are consistent with the observation that cancer cells generally display an elevated rate of transmethylation, which coincides with their increased dependence on a high methylation potential [23]. Supporting this hypothesis, methionine-addicted cancer cells have been shown to over-methylate histone lysines [24,60,61]. Interestingly, this over-methylation of histone lysine marks is unstable in methionine-addicted cancer cells and correlated with malignancy, but remains stable in normal cells or methionine-independent revertants [60,61,64].

It was thus possible that the dependence of cancer cells on exogenous methionine is at least partially caused by misregulation of cellular pathways that depend on efficient methylation steps. Analyses of RNAseq data revealed that the fidelity of splicing is severely affected when methionine-dependent cancer cells are cultured in homocysteine medium, whereas methionine-independent cells better maintain their faithful splicing pattern.

Defects in the *MMACHC* or *MTR* genes affect the cobalamin (Cbl) metabolism (*cblC* or *cblG* defects, respectively), resulting in a reduced activity of methionine synthase [65,66]. Impairment of this enzymatic reaction will lead to the accumulation of homocysteine and the reduced synthesis of methionine, thus disturbing one-carbon metabolism. Interestingly, transcriptomic analyses of *cblC* and *cblG* patient fibroblasts demonstrate significant alterations in pre-mRNA splicing when compared to normal cells [67]. We compared alternative splicing events observed in MDA-MB468 cells cultured with homocysteine for 12 h to published datasets from *cblC* and *cblG* patient-derived fibroblasts (Figure S6). A statistically significant number of shared alternative splicing events were identified. Interestingly, while splicing alterations in MDA-MB468 and HEK293T cells primarily affected genes associated with cell cycle regulation, the common splicing changes in *cblC* and *cblG* patient-derived fibroblasts predominantly involved metabolic genes. This divergence in affected pathways may reflect a heightened sensitivity of tumorigenic cells to alternative splicing in cell cycle-related genes, potentially contributing to their methionine addiction. Alternatively, it may indicate long-term adaptation mechanisms in fibroblasts harboring chronic metabolic defects.

Efficient spliceosome assembly depends on the methylation of Sm proteins, and indeed we found that, unlike most other arginine methylation events, Sm methylation is hypersensitive in methionine-dependent MDA-MB468 cells. In agreement with the notion that loss of Sm methylation reduces the efficiency of the splicing reaction, we observed reduced inclusion of alternative exons in methionine-dependent MDA-MB468 cells. Interestingly, Sm methylation has recently been connected to vulnerability caused by deletion of the p16/ARF tumor suppressor locus [51]. The gene encoding S-methyl-5′-thioadenosine phosphorylase (MTAP), a key enzyme in the methionine salvage pathway, is frequently deleted in cancers due to its proximity to the p16/ARF tumor suppressor locus [68,69]. However, there is no correlation between methionine dependence of cancer and the MTAP status [6], and MTAP deletion has no significant effect on intracellular SAM concentrations [51]. Nevertheless, MTAP deletion has an indirect effect on the cellular methylation potential, because the MTAP substrate methylthioadenosine can act as a competitive inhibitor for methyltransferases [70,71]. Interestingly, the arginine methyltransferase Prmt5, which methylates Sm proteins, appears to be particularly sensitive to inhibition by methylthioadenosine [51,72,73]. While MTAP deletion is not sufficient to affect splicing fidelity, it renders these cancers vulnerable to further inhibition of Prmt5 activity either by genetic mutations or small molecule inhibitors [51,74]. Notably, we demonstrate that methionine-dependent, but not isogenic methionine-independent, cells are hypersensitive to Prmt5 inhibition, which links methionine addiction to Prmt5-mediated pathways. Genetic and chemical genetics results suggest that Prmt5 inhibitor therapy will be most effective for MTAP-deficient tumors [51,72,73]. Our study suggests that methionine-deficient diets could sensitize tumors to Prmt5 inhibition independent of their MTAP status.

In addition to enhancing Prmt5 inhibition, methionine restriction could also augment cancer immunotherapy. The response to immune checkpoint blockade is thought to be determined by neoantigen load. Typically, neoantigen burden is considered mainly mutation-driven [[75], [76], [77]], but recent findings indicate that alterations in splicing also generate meaningful neoantigens that trigger an anti-tumor immune response [78]. Changing the methionine content in the diet may thus offer alternative ways to increase tumor immunogenicity.

Our findings offer insights into the mechanisms underlying methionine addiction of cancer and highlight the role of nutritional influences on splicing fidelity, which may have direct implications for therapeutic strategies. Specifically, we delineate the mechanistic link between Sm protein methylation by Prmt5 and splicing fidelity in the context of methionine addiction. This connection represents one of the potentially many pathways that associate the methionine dependence of cancer with cell proliferation and survival. However, a limitation of this study is the use of relatively high concentrations of dl-homocysteine, which may exert indirect effects specifically on methionine addicted cancer cells. While oxidative stress and ER stress induction were excluded within the timeframe of this study, other physiological consequences of elevated dl-homocysteine levels cannot be ruled out. Future research will aim to further characterize the reported nutrient effects on splicing fidelity and facilitate the development of therapeutic approaches that could be optimized through dietary interventions.

## CRediT authorship contribution statement

**Da-Wei Lin:** Visualization, Methodology, Investigation, Formal analysis. **Francisco G. Carranza:** Visualization, Methodology, Formal analysis, Data curation. **Stacey Borrego:** Validation, Methodology, Investigation, Formal analysis. **Linda Lauinger:** Visualization, Methodology, Investigation, Formal analysis. **Lucas Dantas de Paula:** Visualization, Methodology, Investigation, Formal analysis, Data curation. **Harika R. Pulipelli:** Visualization, Methodology, Investigation, Formal analysis, Data curation. **Anna Andronicos:** Investigation, Funding acquisition, Formal analysis, Data curation. **Klemens J. Hertel:** Writing – review & editing, Writing – original draft, Supervision, Project administration, Funding acquisition, Formal analysis, Data curation, Conceptualization. **Peter Kaiser:** Writing – review & editing, Writing – original draft, Validation, Supervision, Project administration, Funding acquisition, Conceptualization.

## Declaration of competing interest

The authors declare that they have no known competing financial interests or personal relationships that could have appeared to influence the work reported in this paper.

## Data Availability

Data will be made available on request.
