# Supplementary material for: Nutrient control of splice site selection contributes to methionine addiction of cancer
Source: Mol Metab. 2025 Jan 23;93:102103. doi: 10.1016/j.molmet.2025.102103 (PMC11834112; doi:10.1016/j.molmet.2025.102103)
Supplement: Multimedia component 1 [file mmc1.pdf]

# Figure S1

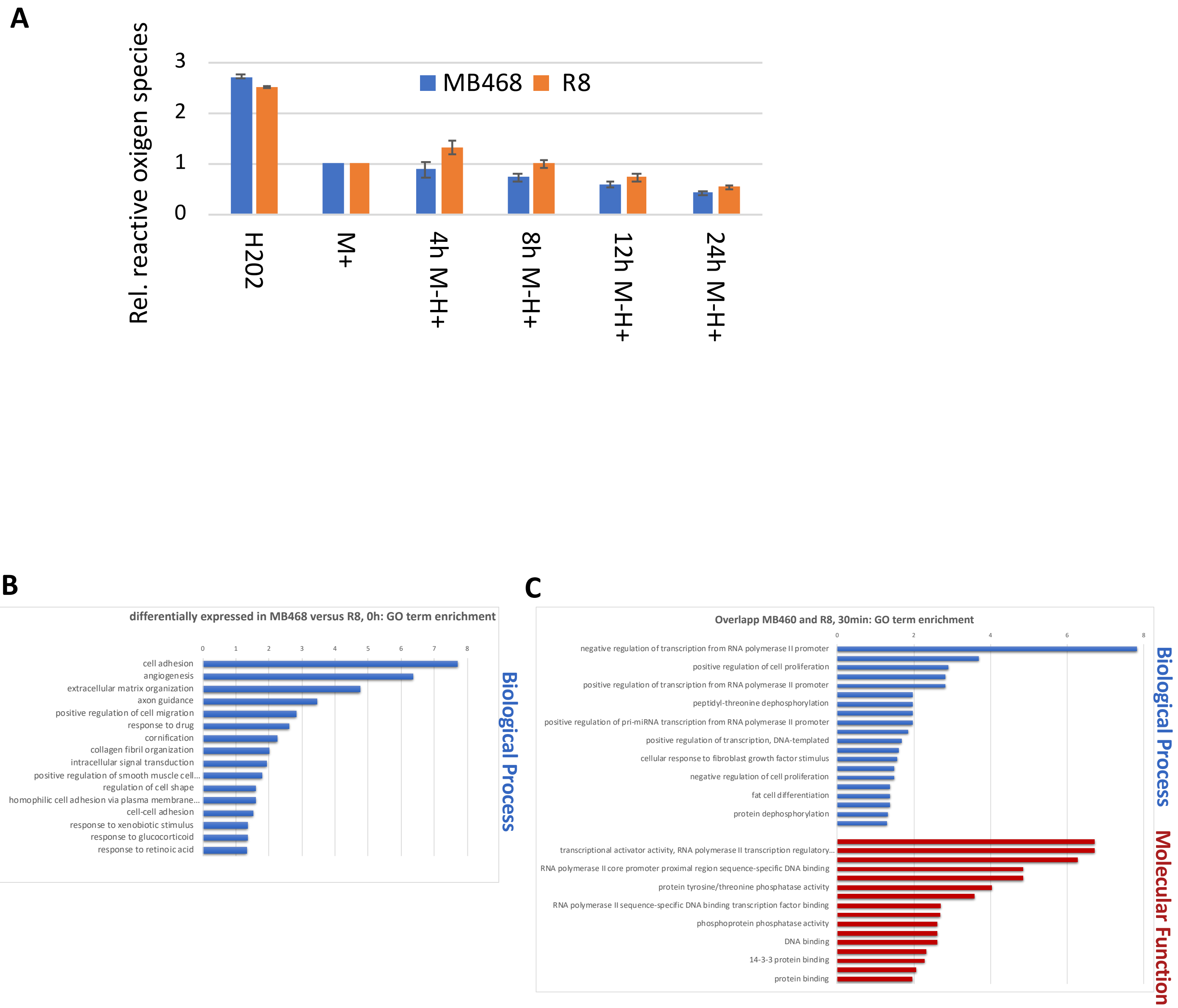
A
Rel. reactive oxigen species
MB468
R8
H202
M+
4h M-H+
8h M-H+
12h M-H+
24h M-H+
B
differentially expressed in MB468 versus R8, 0h: GO term enrichment
cell adhesion
angiogenesis
extracellular matrix organization
axon guidance
positive regulation of cell migration
response to drug
cornification
collagen fibril organization
intracellular signal transduction
positive regulation of smooth muscle cell...
regulation of cell shape
homophilic cell adhesion via plasma membrane...
cell-cell adhesion
response to xenobiotic stimulus
response to glucocorticoid
response to retinoic acid
Biological Process
C
Overlapp MB460 and R8, 30min: GO term enrichment
negative regulation of transcription from RNA polymerase II promoter
positive regulation of cell proliferation
positive regulation of transcription from RNA polymerase II promoter
peptidyl-threonine dephosphorylation
positive regulation of pri-miRNA transcription from RNA polymerase II promoter
positive regulation of transcription, DNA-templated
cellular response to fibroblast growth factor stimulus
negative regulation of cell proliferation
fat cell differentiation
protein dephosphorylation
transcriptional activator activity, RNA polymerase II transcription regulatory...
RNA polymerase II core promoter proximal region sequence-specific DNA binding
protein tyrosine/threonine phosphatase activity
RNA polymerase II sequence-specific DNA binding transcription factor binding
phosphoprotein phosphatase activity
DNA binding
14-3-3 protein binding
protein binding
Biological Process
Molecular Function

Figure S2

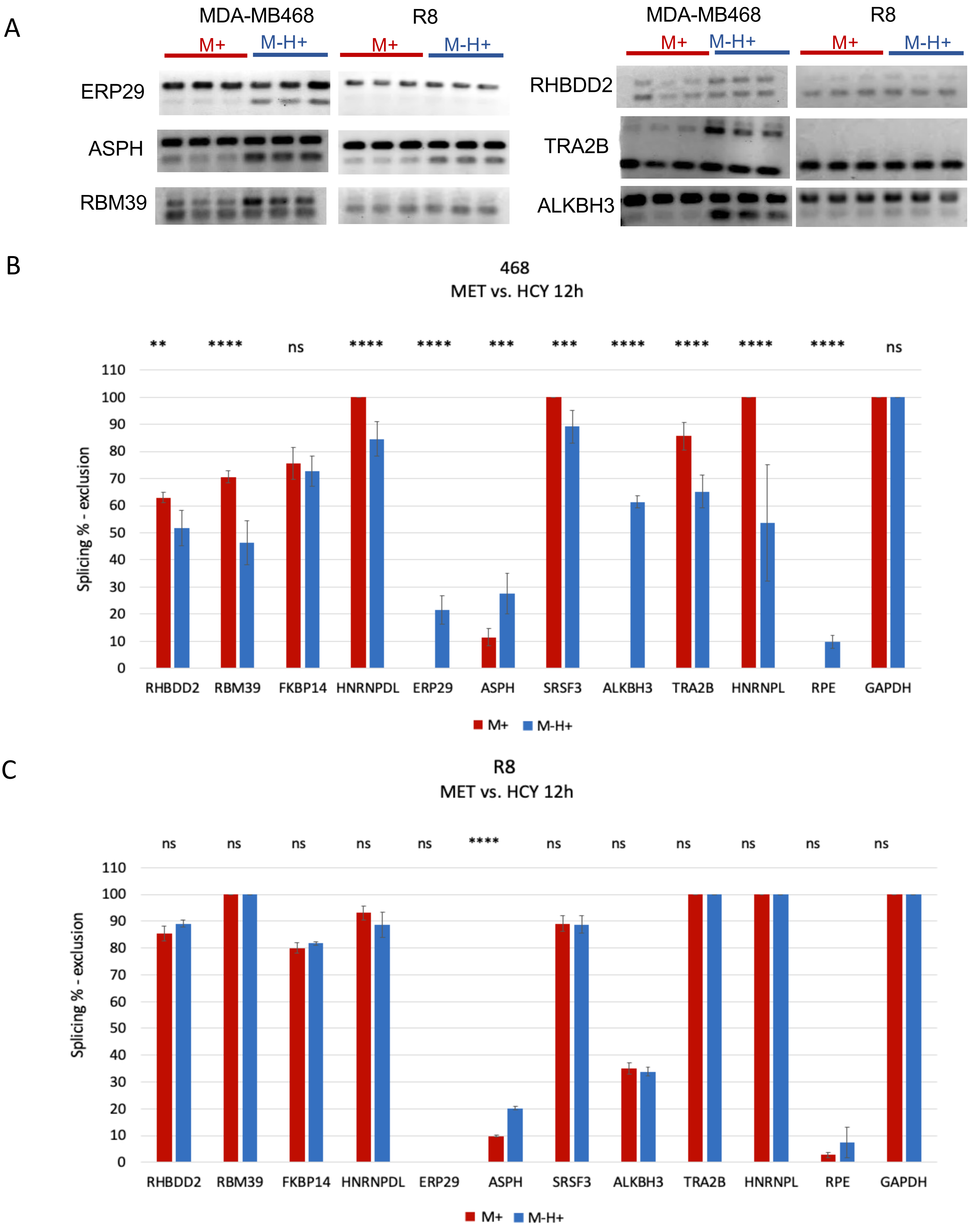
A
MDA-MB468
R8
M+
M-H+
ERP29
ASPH
RBM39
RHBDD2
TRA2B
ALKBH3
B
468
MET vs. HCY 12h
** **** ns **** **** *** *** **** **** **** **** ns
Splicing % - exclusion
0 10 20 30 40 50 60 70 80 90 100 110
RHBDD2 RBM39 FKBP14 HNRNPDL ERP29 ASPH SRSF3 ALKBH3 TRA2B HNRNPL RPE GAPDH
M+ M-H+
C
R8
MET vs. HCY 12h
ns ns ns ns ns **** ns ns ns ns ns ns
Splicing % - exclusion
0 10 20 30 40 50 60 70 80 90 100 110
RHBDD2 RBM39 FKBP14 HNRNPDL ERP29 ASPH SRSF3 ALKBH3 TRA2B HNRNPL RPE GAPDH
M+ M-H+

# Figure S3

## MB468 0vs720 SE

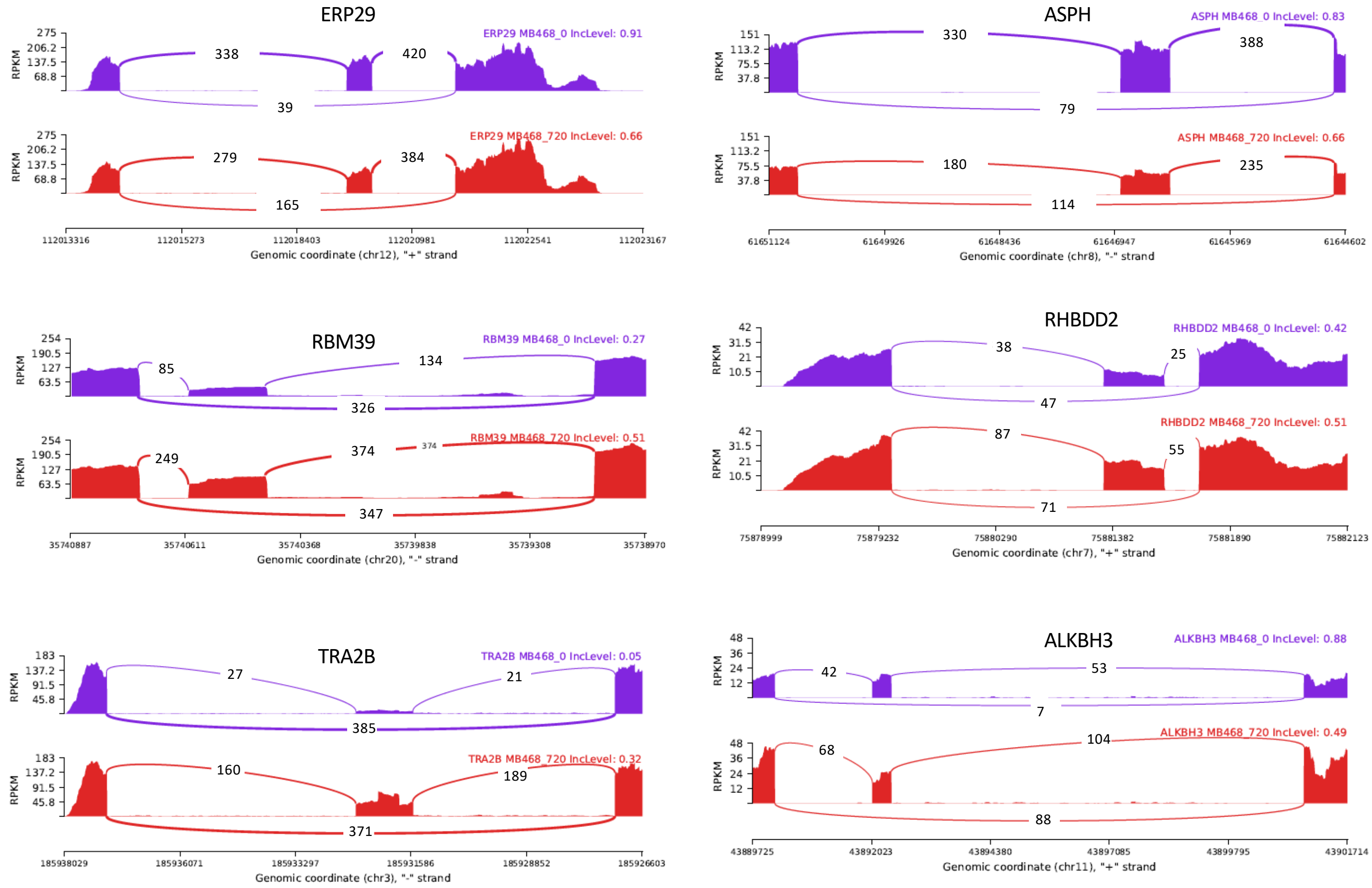


## R8 0vs720 SE

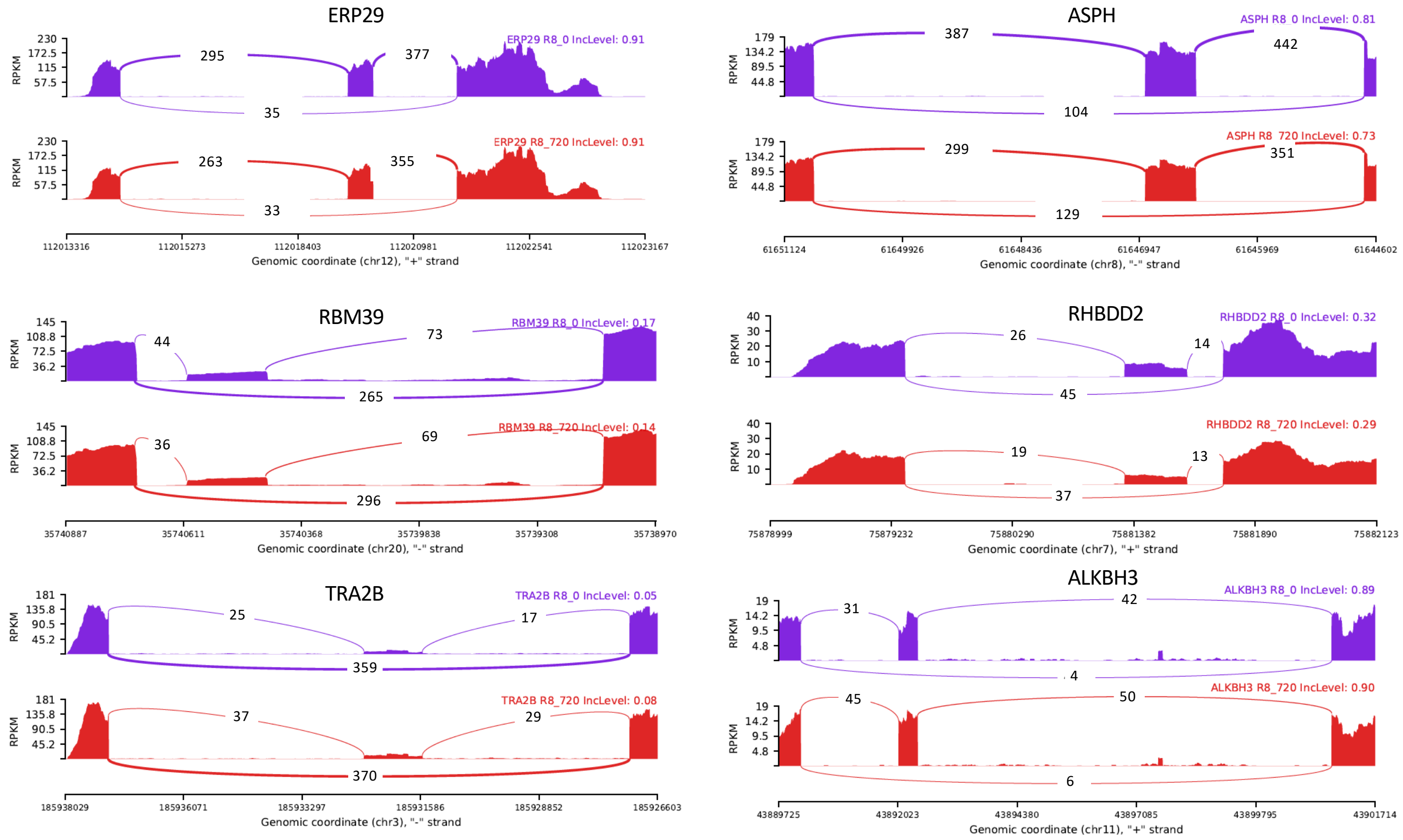

**Figure S4**

A

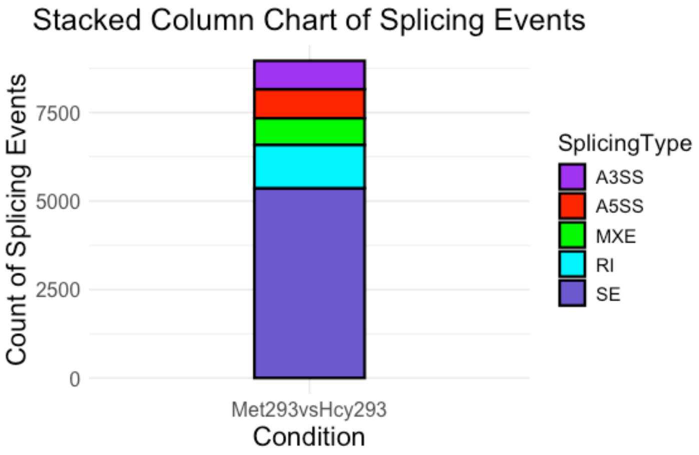


B

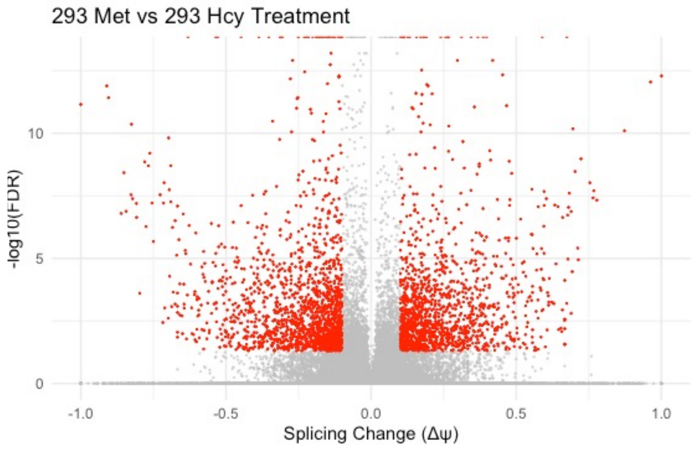


C

Comparison between 468s after 12 hrs Hcy and 293Ts after 12 hrs Hcy

All exons with FDR <0.05 and >10% inclusion gain in Hcy

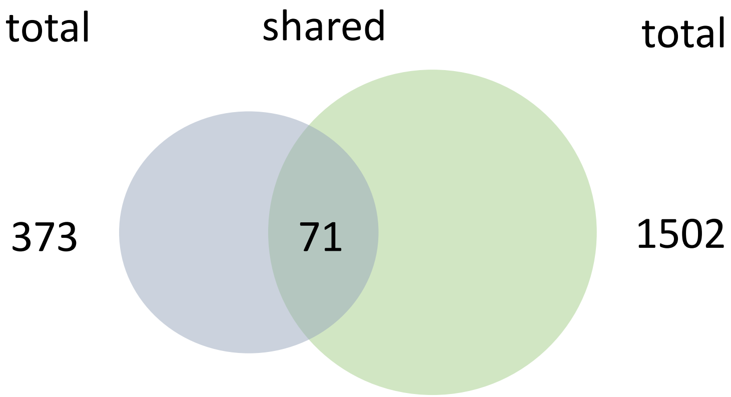


Significance of overlap: p-value = 9.4 e-38

D

Gene enrichment of AS events upon methionine restriction

293T cells after 12 hrs Hcy

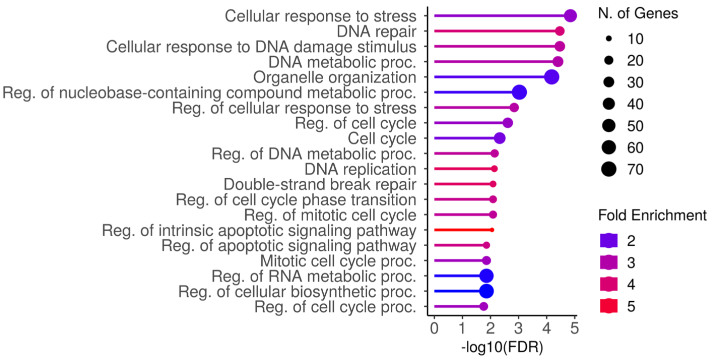


Overlap between 468 and 293T cells after 12 hrs Hcy

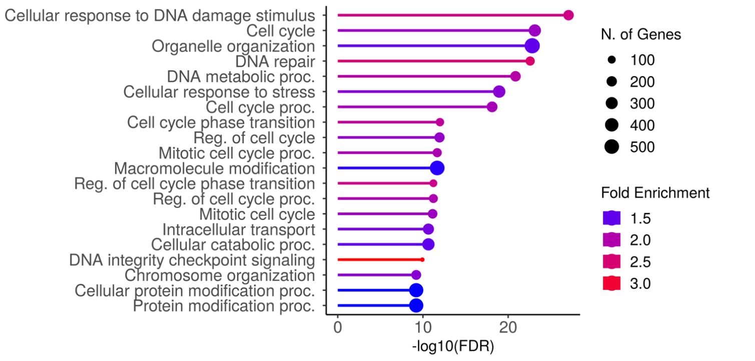

**Figure S5**

A

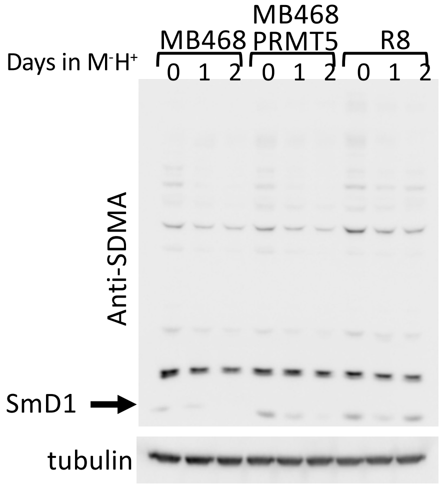

Days in M-H+
MB468
MB468 PRMT5
R8
0 1 2 0 1 2 0 1 2
Anti-SDMA
SmD1
tubulin


B

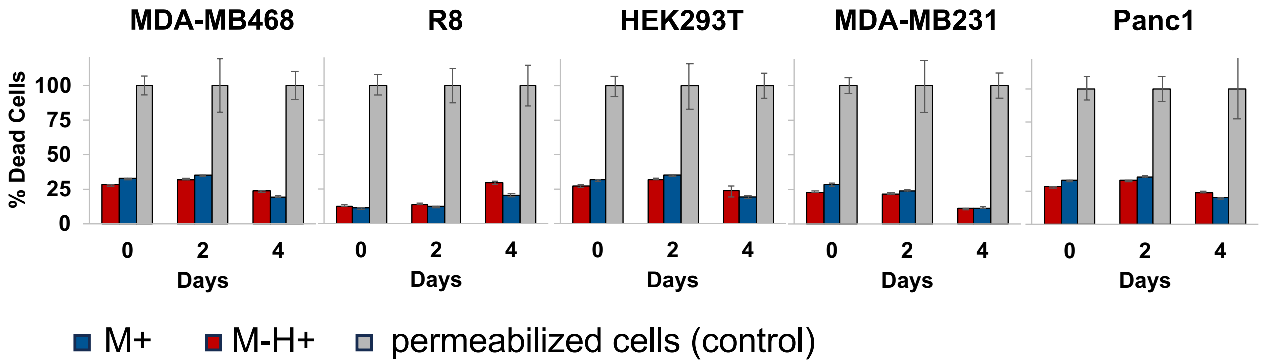

MDA-MB468
R8
HEK293T
MDA-MB231
Panc1
% Dead Cells
100
75
50
25
0
0 2 4
Days
M+
M-H+
permeabilized cells (control)

**Figure S6**

## Overlap of genes with alternative splicing events
## 468s after 12 hrs Hcy vs *cblC* or *cblG* metabolic defect

### *cblC* defect all events within a gene  with FDR <0.05

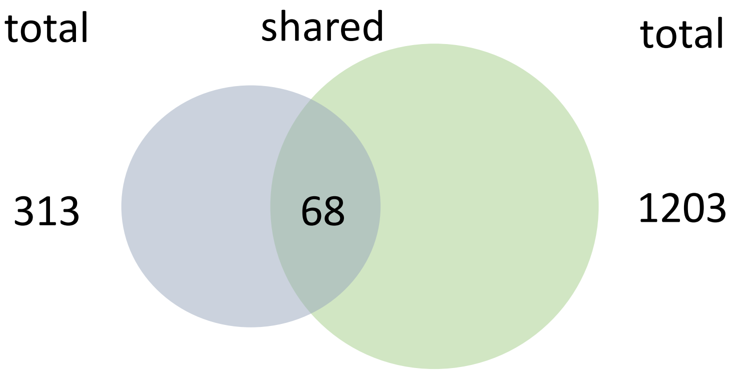


Significance of overlap: p-value = 4 e-47

### *cblG* defect all events within a gene with FDR <0.05

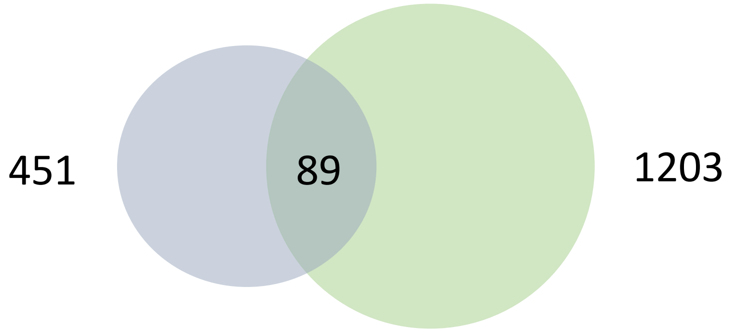


Significance of overlap: p-value = 1.2 e-57

**Figure legends for supplemental figures**

**Figure S1. Comparison of MB468 and R8 in methionine medium and switch to homocysteine media.** (A) Oxidative stress was compared between MDA-MB468 and R8 cells upon switch to homocysteine medium using a FACS-based CM-H2DCFDA assay. Cells treated with $H_2O_2$ serve as a positive control. (B) Ontology terms of differentially expressed genes between MDA-MB468 and R8 cells in methionine medium. (C) Ontology terms of genes responding to 30 min methionine stress in both MDA-MB468 and R8 cells.

**Figure S2. PCR verification of differential alternative splicing events upon methionine restriction.** (A) Representative agarose gel electrophoresis images highlighting differential splicing events upon methionine/homocysteine switch in MBA-MD468 and R8 cells. (B and C) quantitation of the data shown in (A). GAPDH serves as a negative control.

**Figure S3. Sashimi plot of differential alternative splicing events upon methionine restriction.** Representative Sashimi plots were created for alternative splicing events identified by transcriptomics and verified by RT-PCR (Fig. S2). The top half displays alternative splicing differences upon methionine/homocysteine switch in MBA-MD468 cells. The bottom half displays the same analysis for R8 cells. Purple plots illustrate exon inclusion levels in the presence of methionine. Red plots represent exon inclusion levels after 720 min of methionine withdrawal.

**Figure S4. Methionine restriction in HEK293T cells impacts splicing fidelity.** (A) A stacked bar graph displaying the number of alternative splicing events observed in HEK293T cells after 720 min into $M^-H^+$ medium shift. SE: skipped exons; IR: intron retention; MXE: mutually exclusive exons; A5SS: alternative 5’ splice sites; A3SS: alternative 3’ splice sites. (B) Volcano plot displaying the difference in the fraction spliced of skipped exon events in 293T cells upon switching to homocysteine growth media. Red indicates all alternative exon inclusion events with >10% change and FDR <0.05. (C) Overlap of methionine-restriction sensitive exons between MDA-MB468 cells and 293T cells upon methionine restriction. (D) Gene ontology analysis of genes harboring methionine-restriction sensitive exons in 293T cells (left panel), and genes with overlapping alternative splicing events between MDA-MB468 cells and HEK293T cells upon methionine restriction (right panel).

**Figure S5. Synthetic dimethyl arginine modification and oxidative stress induction upon switch to homocysteine medium.** (A) Lower exposure of SDMA blot shown in figure 6A. (B) Cell viability assay (CellTox Green) was used to assess the fraction of viable cells upon shift to homocysteine medium for up to 4 days. Permeabilized cells were used as a control to assess maximum cell death (n= 6, mean +/- SE).

**Figure S6. Splicing overlap analysis of MDA-MB468 methionine restriction and *cblC* or *cblG* metabolic defects.** Defects in the *MMACHC* or *MTR* genes affect Cbl metabolism (*cblC* or *cblG* defects, respectively), resulting in the accumulation of homocysteine and the reduced synthesis of methionine. An analysis of available transcriptomic data from *cblC* or *cblG* patient fibroblast cell lines (Rashka et al., 2020) was used to identify common gene targets of alternative splicing between MDA-MB468 methionine restriction and *cblC* or *cblG* defects. The top Venn diagram displays the overlap between MDA-MB468 methionine restriction and *cblC*, the bottom diagram displays the overlap between MDA-MB468 methionine restriction and *cblG*.

## Supplemental references

Rashka, C., Hergalant, S., Dreumont, N., Oussalah, A., Camadro, J.-M., Marchand, V., et al., 2020. Analysis of fibroblasts from patients with cblC and cblG genetic defects of cobalamin metabolism reveals global dysregulation of alternative splicing. Human Molecular Genetics 29(12): 1969–85, Doi: 10.1093/hmg/ddaa027.
